# Visualizing complex healthcare disparities: proof of concept for representing a cyclical continuum of care model for a retrospective cohort of patients with musculoskeletal infections

**DOI:** 10.1186/s12891-021-04358-7

**Published:** 2021-05-21

**Authors:** Martha L. Carvour, Allyssa Chiu, Kimberly Page

**Affiliations:** 1 Division of Epidemiology, Biostatistics, and Preventive Medicine; Department of Internal Medicine, University of New Mexico Health Sciences Center, 1 University of New Mexico; MSC 10-5550, Albuquerque, NM 87131 USA; 2 Division of Infectious Diseases; Department of Internal Medicine, University of New Mexico, 1 University of New Mexico; MSC 10-5550, Albuquerque, NM 87131 USA; 3 Division of Infectious Diseases; Department of Internal Medicine, University of Iowa Carver College of Medicine, 200 Hawkins Drive, Iowa City, IA 52242 USA

**Keywords:** Healthcare disparities, Health equity, Healthcare access, Musculoskeletal infections, Amputation, Data visualization

## Abstract

**Background:**

Care continuum models (also known as care cascade models) are used by researchers and health system planners to identify potential gaps or disparities in healthcare, but these models have limited applications to complex or chronic clinical conditions. Cyclical continuum models that integrate more complex clinical information and that are displayed using circular data visualization tools may help to overcome these limitations. We performed proof-of-concept cyclical continuum modeling for one such group of conditions—musculoskeletal infections—and assessed for racial and ethnic disparities across the complex care process related to these infections.

**Methods:**

Cyclical continuum modeling was performed in a diverse, retrospective cohort of 1648 patients with musculoskeletal infections, including osteomyelitis, septic arthritis, and/or infectious myositis, in the University of New Mexico Health System. Logistic regression was used to estimate the relative odds of each element or outcome of care in the continuum. Results were visualized using circularized, map-like images depicting the continuum of care.

**Results:**

Racial and ethnic disparities differed at various phases in the care process. Hispanic/Latinx patients had evidence of healthcare disparities across the continuum, including diabetes mellitus [odds ratio (OR) 2.04, 95% confidence interval (CI): 1.61, 2.60 compared to a white non-Hispanic reference category]; osteomyelitis (OR 1.28, 95% CI: 1.01, 1.63); and amputation (OR 1.48; 95% CI: 1.10, 2.00). Native American patients had evidence of disparities early in the continuum (diabetes mellitus OR 3.59, 95% CI: 2.63, 4.89; peripheral vascular disease OR 2.50; 95% CI: 1.45, 4.30; osteomyelitis OR 1.43; 95% CI: 1.05, 1.95) yet lower odds of later-stage complications (amputation OR 1.02; 95% CI: 0.69, 1.52). African American/Black non-Hispanic patients had higher odds of primary risk factors (diabetes mellitus OR 2.70; 95% CI: 1.41, 5.19; peripheral vascular disease OR 4.96; 95% CI: 2.06, 11.94) and later-stage outcomes (amputation OR 2.74; 95% CI: 1.38, 5.45) but not intervening, secondary risk factors (osteomyelitis OR 0.79; 95% CI: 0.42, 1.48).

**Conclusions:**

By identifying different structural and clinical barriers to care that may be experienced by groups of patients interacting with the healthcare system, cyclical continuum modeling may be useful for the study of healthcare disparities.

## Background

Population health scientists and health system administrators seek to understand the health needs of large populations and identify population-level or system-level interventions that benefit diverse groups of patients while still ensuring effective stewardship of the finite resources of healthcare or public health systems. Such discussions may rely on the timely, effective combination of population data, clinical knowledge, and infrastructural insight. Data visualization methods that represent population-level or system-level healthcare outcomes data may help to track the engagement of diverse populations of patients with evidence-based care in these complex systems and may, in turn, inform efforts to systematically enhance access to evidence-based care (e.g., by identifying and reducing structural barriers at steps in the healthcare process or by identifying and improving the steps with the most frequent gaps in care).

Traditionally, “continuum” or “cascade” models have been used to depict population-level or system-level health outcomes data by defining, measuring, and depicting sequential elements or outcomes of care for specific health conditions and tracking the occurrence of those elements or outcomes within affected populations [1–10]. Continuum or cascade models typically represent a series of steps in the process of care—such as diagnosis, treatment, and cure—by depicting the number or proportion of people in the population completing each step in the cascade or continuum using a bar chart or frequency distribution. Because these models depict the process of care on the population-level or health system-level, they can be used to identify gaps in care that occur across a population or that differ across subgroups in a population [3, 11–15] when the visualized data are stratified by another factor of interest (e.g., a sociodemographic factor that may be associated with disparities in healthcare access or utilization). Unfortunately, since traditional cascade or continuum models typically represent a series of singular, hierarchical steps in the care process [1, 3, 6–10], these models are not well suited to depict the elements or outcomes of care for more complex or chronic conditions [14, 15]. To address this limitation, we previously proposed a circularized—or cyclical—adaptation of traditional continuum models for the study of complex or chronic clinical processes. Like traditional continuum models, cyclical continua may still permit visual comparisons of patients’ experiences with the healthcare system across subgroups in a population [15] when the visualized data are stratified by a factor of interest (e.g., a sociodemographic variable).

Previously, in a cohort of patients with serious musculoskeletal infections at the University of New Mexico (UNM) Hospital, we found evidence of significant racial and ethnic disparities in infection-related amputation—a late-stage outcome in the healthcare process [16]. We found that these disparities could not be fully explained by disparities in diabetes diagnosis or diabetes control or by corresponding disparities in access to surgical procedures overall [16]. To provide further context for these disparities within the process of care before, during, and after a diagnosis of a musculoskeletal infection, we performed a proof-of-concept cyclical continuum analysis for the UNM cohort, using a cyclical continuum model of eight key elements and outcomes of care relevant to serious musculoskeletal infections. We report those proof-of-concept findings here. For this study, we hypothesized that racial and ethnic disparities leading up to the amputation outcome would differ across the process of care and that these disparities would be visually discernible using cyclical continuum modeling.

## Methods

### Cohort and setting

We performed a proof-of-concept cyclical continuum analysis in a retrospective cohort of musculoskeletal infections in the UNM Health System. Inclusion criteria for the cohort were as follows: adult patients (≥18 years of age) who were hospitalized at UNM and diagnosed with one or more serious musculoskeletal infection (including osteomyelitis, septic arthritis, and/or infectious myositis) between January 1, 2010 and December 31, 2015.

Clinical and sociodemographic data for each patient were obtained from the electronic medical record for each patient, including International Classification of Diseases (versions 9 and 10) codes for each diagnostic outcome. Further descriptions of the cohort, and the power calculations underlying its formation, have been published previously [16].

All data were extracted and deidentified by the UNM Clinical and Translational Sciences Data Warehouse before transmission to the research team. The UNM Institutional Review Board reviewed and approved this study.

### Cyclical continuum

**Fig. 1:**
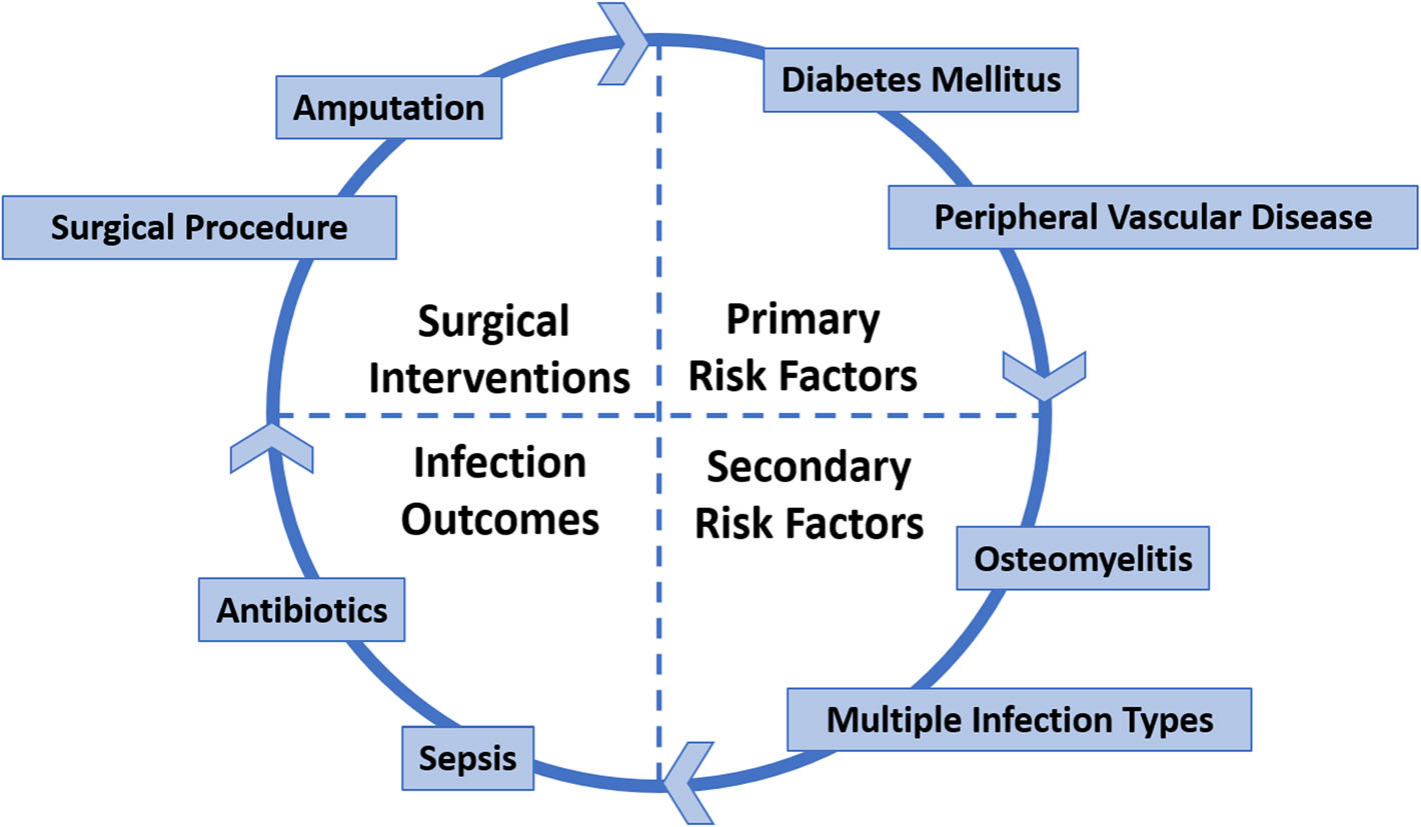
Cyclical continuum model of musculoskeletal infection, showing four general clinical phases with two elements or outcomes of interest per phase

The cyclical model for this proof-of-concept analysis included four clinical phases, each of which had two defined elements or outcomes of care which corresponded to variables in the cohort dataset (Fig. 1). The phases of care ranged from primary risk factors for limb loss (measured elements/outcomes: diabetes mellitus and peripheral vascular disease) and secondary risk factors for limb loss (measured elements/outcomes: osteomyelitis and two or more musculoskeletal infection types) to infection-related outcomes (measured elements/outcomes: sepsis and antibiotic use) and surgical interventions (measured elements/outcomes: any surgical procedure, including amputation, and any amputation procedure) (Fig. 1). Patients were classified as having diabetes mellitus if one or more of the following criteria were present: any diagnostic code for diabetes (including any type or complication of diabetes), a maximum hemoglobin A1c ≥6.5% (48 mmol/mol), or a prescription for any medication used to treat diabetes. Patients were classified as having peripheral vascular disease, osteomyelitis, sepsis, surgical procedures, or amputations if any corresponding diagnostic or procedure code was recorded. To capture the initial management phase for the musculoskeletal infections, surgical procedures and amputations occurring at or near the anatomic site of the infection within the first 3 months of the infection diagnosis were included. To capture the relative complexity or extent of the initial musculoskeletal infection, the outcome depicting multiple musculoskeletal infection types was defined as having two or more of the musculoskeletal infection diagnoses—osteomyelitis, septic arthritis, and infectious myositis. Antibiotic use was defined as prescription of any systemic antimicrobial medication.

### Data analysis

In traditional continuum models that apply absolute measures, such as counts or frequencies, the first element of care or first clinical outcome in a continuum (i.e., diabetes as shown in Fig. 1) might serve as a denominator for all subsequent elements. In our case, however, patients had already been selected into the existing cohort on the basis of a secondary risk factor for limb loss (i.e., one or more musculoskeletal infections as outlined in the inclusion criteria in “Cohort and Setting” above) and not based on a primary risk factor for limb loss (e.g., diabetes mellitus). Thus, we opted to represent all eight elements or outcomes in the continuum model (Fig. 1)—from primary risk factors for limb loss through surgical outcomes—using relative measures instead of absolute measures. This approach would permit relatively uniform comparisons across elements and outcomes in the cycle, including those which preceded the element on which cohort selection was based.

For each element or outcome of care shown in Fig. 1, we calculated odds ratios (ORs) and 95% confidence intervals (CIs), adjusted for age and sex using multivariable logistic regression, in each racial and ethnic group and projected these values onto a cyclical continuum visual on a natural log scale. Since we were interested in evaluating potential disparities across the care process, the racial and ethnic group with the lowest odds of the first primary risk factor from Fig. 1 (diabetes mellitus) in the cohort was used as the reference category for all ORs and CIs. To further characterize the cohort, age and sex were also compared across the racial and ethnic categories using ANOVA and chi-square testing, respectively. All statistical analyses were performed using SAS (SAS Institute, Inc.; Cary, North Carolina). *P*-values < 0.05 were considered statistically significant.

### Data visualization

Proof-of-concept circular data visualization was performed using the radar mapping tool in Microsoft Excel. Maps were generated for each of four racial and ethnic groups. The adjusted OR for each element of care was displayed, compared to the reference category with the lowest odds of the first primary risk factor in the continuum (diabetes mellitus). Asterisks were used to signify statistical significance where the 95% CI excluded 1.00.

## Results

### Characteristics

The cohort (*N* = 1648) was diverse with respect to sex (*N* = 522 women, 31.7%) and race/ethnicity (Table 1). Age and sex were chosen a priori as factors for adjustment of each outcome in the cyclical continuum model. There were statistically significant differences in age (*p* < 0.0001 from ANOVA, Table 1) across racial/ethnic groups in the cohort, but this was not observed for sex (*p* = 0.22 from a chi-square test). Native American patients tended to be younger than patients in other groups (Table 1).

**Table 1:** Average age of cohort members by race/ethnicity

	N (%) of Patients in Cohort (Total ***N*** = 1648)	Mean (Median) Age +/− Standard Deviation, in Years
Hispanic or Latinx	662 (40.2%)	52.3 (53.0) +/− 15.8
White Non-Hispanic	577 (35.0%)	56.3 (58.0) +/− 14.3
Native American	292 (17.7%)	50.9 (51.0) +/− 14.6
Two or More Races, Asian/Pacific Islander, or Other Race/Ethnicity	74 (4.5%)	54.6 (58.0) +/− 16.3
African American/Black Non-Hispanic	43 (2.6%)	53.4 (55.0) +/− 13.7

### Cyclical continua

**Fig. 2:**
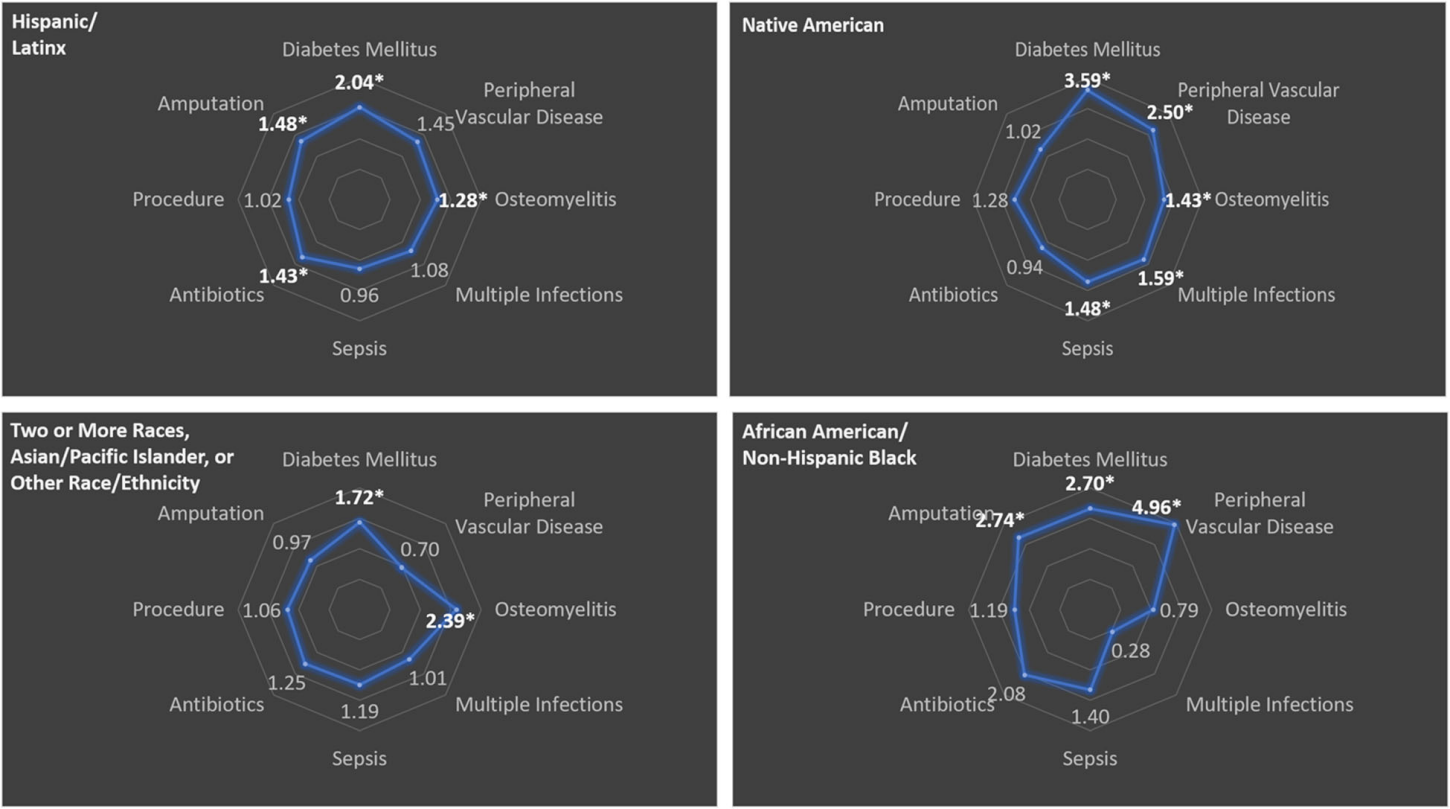
Cyclical model results for each of four racial and ethnic groups compared to the reference category (white non-Hispanic patients) with the lowest odds of the index outcome (diabetes mellitus) in the cycle. Values shown are odds ratios for each outcome (e.g., diabetes mellitus), compared to the reference category and adjusted for age and sex. Asterisks (*) signify statistical significance, where the 95% confidence interval excludes 1.00. All panels are shown on a natural log scale

Cyclical continuum maps for each racial and ethnic group are shown in Fig. 2. These correspond to the tabular data shown in Table 2. The panels in Fig. 2 reveal different patterns in the process of care for each of four racial and ethnic groups, compared to the white non-Hispanic reference category (that is, the category with the lowest odds of the index outcome of diabetes). In general, Hispanic/Latinx patients had increased odds of some primary and secondary risk factors as well as amputation, whereas Native American patients had significantly higher odds of all primary and secondary risk factors and sepsis yet lower odds of amputation. African American/Black non-Hispanic patients had high odds of both primary risk factors, low odds of both secondary risk factors (including osteomyelitis), and yet the highest odds of undergoing an amputation.

**Table 2:** Relative odds for each element or outcome of care for patients in the cohort

	Hispanic/Latinx	Native American	African American/Non-Hispanic Black	Two or More Races, Asian/ Pacific Islander, or Other Race/ Ethnicity
Diabetes mellitus	2.04 (1.61, 2.60)*	3.59 (2.63, 4.89)*	2.70 (1.41, 5.19)*	1.72 (1.03, 2.87)*
Peripheral vascular disease	1.45 (0.90, 2.34)	2.50 (1.45, 4.30)*	4.96 (2.06, 11.94)*	0.70 (0.21, 2.37)
Osteomyelitis	1.28 (1.01, 1.63)*	1.43 (1.05, 1.95)*	0.79 (0.42, 1.48)	2.39 (1.30, 4.39)*
Multiple infections	1.08 (0.79, 1.48)	1.59 (1.10, 2.29)*	0.28 (0.07, 1.19)	1.01 (0.51, 2.00)
Sepsis	0.96 (0.74, 1.24)	1.48 (1.09, 2.03)*	1.40 (0.72, 2.74)	1.19 (0.70, 2.03)
Antibiotics	1.43 (1.00, 2.04)*	0.94 (0.62, 1.41)	2.08 (0.63, 6.88)	1.25 (0.58, 2.72)
Surgical procedure	1.02 (0.80, 1.30)	1.28 (0.93, 1.76)	1.19 (0.60, 2.38)	1.06 (0.62, 1.80)
Amputation	1.48 (1.10, 2.00)*	1.02 (0.69, 1.52)	2.74 (1.38, 5.45)*	0.97 (0.50, 1.89)

Note: Data shown are odds ratios (95% confidence intervals) adjusted for age and sex compared to the reference category (white non-Hispanic patients) with the lowest odds of the index outcome (diabetes mellitus). These data correspond with the results projected onto the circular visual in Fig. 2. *Statistically significant (95% confidence interval excludes 1.00 or *p* < 0.05)

## Discussion

Continuum and cascade models have previously been applied to measure important elements in the healthcare process occurring on the population level for both infectious and non-infectious conditions [1–10], yet traditional hierarchical continua have limited applications to conditions which—like musculoskeletal infections—may be chronic or recurrent (e.g., a recurring foot infection related to a non-healing ulcer in a patient with diabetes) or which involve multiple, complex endpoints of interest in the clinical care process (e.g., interrelated, yet often non-hierarchical, risk factors for limb loss, such as diabetes and peripheral vascular disease) [15]. Circularized models that preserve the benefits of traditional continua, while addressing the complexities of clinical management for other complex or chronic conditions, may expand the utility of the continuum approach.

In this report, we provide proof-of-concept, circularized representations of care continua for a cohort of patients with musculoskeletal infections at a single academic medical center. The results displayed in Fig. 2 suggest some important benefits of this visual approach. Like traditional continuum models using frequency distributions or other similar graphics, the circularized image condenses complex data into a single visual tool—a map-like image of the care process—that may aid discussions about the process of care with constituents across the health system. Such imagery may provide a more accessible, informative representation of the care process to population health scientists, health system administrators, clinical providers, and patients than tabular representations of the same data (as shown in Table 2) and may inform discussions about disparities in healthcare access or healthcare outcomes. In the future, more complex circular visualization techniques may be useful in exploring the utility of circularized continua as a population health tool. However, we note that the ease of navigating a straightforward, Excel-based tool to generate visual map-like projections of the care process, as we have done in this proof-of-concept analysis, may still be helpful for many sites or centers without access to specialized informatics software.

As Fig. 2 also demonstrates, stratifying the continuum on key variables of interest may reveal important patterns across the process of care that might otherwise be difficult to appreciate with representations of singular or hierarchical endpoints. In this case, the maps appear to show different patterns of engagement with or access to the healthcare system for different patient subgroups across the four general phases of care from primary risk factors for limb loss through later-stage interventions, such as amputation. In this cohort, Hispanic/Latinx patients appeared to encounter disparities across multiple phases of care, including amputation, whereas Native American patients had evidence of disparities early in the care process that did not clearly translate into increased odds of amputation. Meanwhile, African American/Black non-Hispanic patients had increased odds of primary risk factors and limb loss, without significantly increased odds of secondary risk factors.

Clinically, these results may have important implications. If patient groups experience different structural or clinical barriers to evidence-based care, then improving access to care and advancing equitable healthcare outcomes may require different structural or clinical interventions across the continuum of care. Although these results constitute a preliminary assessment of patients’ experiences within our health system, cyclical continuum modeling may help to guide the planning or selection of interventions to improve care—or improve access to care—by depicting common barriers experienced across subgroups or, conversely, by identifying barriers that are likely to impact specific subgroups. In addition to modeling the experiences of patients across sociodemographic subgroups, cyclical continuum subgroup stratification could also be considered for other variables, including clinically relevant classifications (e.g., disease severity), distinct centers or sites within a health system (e.g., various clinics or hospital units), or interventions performed on the patient or population level (e.g., pre- and post-intervention time points).

In this study, we applied relative measures to represent the experiences of patients with each element of care in the continuum. Although this approach also differs from many traditional continuum models, it demonstrates another potential advantage of cyclical modeling. The use of relative measures in this context permits researchers to measure elements or outcomes of care from any “perspective” of interest in the continuum (e.g., musculoskeletal infection) rather than select a single primary element as the benchmark by which all successive steps in the continuum are measured. Likewise, this approach does not require that all elements of care must occur for each patient. In fact, clinically indicated outcomes (e.g., antibiotic use) and clinically undesirable outcomes (e.g., complications such as sepsis) can be included in the same model to more comprehensively represent patients’ experiences and permit comparison of competing outcomes which may provoke or preclude other elements in the model.

In the future, cyclical continuum models and circular data visualization tools may be further developed for use with other clinical scenarios and more advanced informatics tools, such as larger databases and more complex circular visualization software. With this proof-of-concept analysis, we encountered several important methodological questions and limitations that should be considered in such future work. First, larger scale applications of the cyclical continuum concept will require careful definition of the specific phases and elements or outcomes of care (including the competing risks or endpoints these may represent) and the specific populations or subpopulations of interest. Further exploration of the complementary role of absolute and relative measures in cyclical continuum modeling is also needed. Similarly, if relative measures are employed, the basis for selecting one or more “perspectives” from which each cycle is viewed, as well as the conventions for choosing reference categories for stratified analyses, is worthy of ongoing deliberation. Finally, the methods for visually depicting statistical output, including statistical error, on cyclical maps should be further evaluated and optimized.

We acknowledge that these methodological questions cannot be fully addressed by our proof-of-concept analysis. Other limitations of our study include the use of data for only a single academic health system and a single time window. Although this scenario may reflect a potential practical application of cyclical models—that is, for informing individual health systems about gaps or disparities in the care process—the utilities and challenges of similar models in different systems over different time points should be evaluated in order to refine the methods used. Our analysis also does not include any formal presentation of the utility of cyclical continua for communicating findings with clinical providers, health system administrators, or patients. We have informally found this cyclical map-like data visualization technique to appeal to interdisciplinary clinical providers at our center; but this is worthy of further, formal evaluation.

## Conclusions

In this report, we demonstrate that disparities in health outcomes can vary across patient subgroups along different segments of the clinical care process. We offer preliminary evidence that cyclical continuum modeling and circular data visualization may constitute useful tools for identifying, depicting, and addressing healthcare disparities on the system-level or population-level. We also outline several important steps needed to support full-scale implementation of cyclical continuum modeling as a tool for visualizing complex healthcare disparities. Population health scientists and health system planners may consider cyclical models when standard, hierarchical models cannot be applied, as in the case of complex, chronic, or recurrent clinical conditions of interest on the population level.

## Data Availability

Data are not publicly available due to the authors’ security agreement with the University of New Mexico Clinical and Translational Sciences Data Warehouse. External review of the data used in this study would require approval of the University of New Mexico Clinical and Translational Sciences Data Warehouse and the University of New Mexico Institutional Review Board. The corresponding author may be contacted for inquiries about this process.

## Abbreviations

UNM: University of New Mexico
OR: Odds ratio
CI: Confidence interval
ANOVA: Analysis of variance
